# Social comparison effects on students’ cognitive anxiety, self-confidence, and performance in Chinese composition writing

**DOI:** 10.3389/fpsyg.2022.1060421

**Published:** 2022-11-15

**Authors:** Jon-Chao Hong, Kai-Hsin Tai, Ming-Yueh Hwang, Chia-Yin Lin

**Affiliations:** ^1^Chinese Language and Technology Center, National Taiwan Normal University, Taipei, Taiwan; ^2^Global Program in Interdisciplinary Studies, Soochow University, Taipei, Taiwan; ^3^Continuing Education Master’s Program of Creativity Development, National Taiwan Normal University, Taipei, Taiwan

**Keywords:** cognitive anxiety in writing, downward comparison, self-confidence in writing, social comparison, upward comparison

## Abstract

Social comparison is a mind-altering determinant that affects students’ learning behavior. To understand the effect, three instructional approaches to teaching Chinese writing skills were designed and implemented in this study: (1) The No Comparison Group (NCG): students were asked to complete compositions on their own; (2) The Upward Comparison Group (UCG): superior composition examples were provided and the students were asked to write compositions on the same topics; and (3) The Downward Comparison Group (DCG): inferior examples were provided for students to critique. Taiwanese junior high school ninth graders participated in three groups, and wrote compositions on six themes. The results revealed that the Chinese composition writing (CCW) skills of the students in the UCG and DCG improved significantly more than those of the students in the NCG. Composition-prompted cognitive anxiety in the DCG declined substantially. The results imply that adopting upward and downward comparisons for students to practice Chinese composition is worth adopting in writing lessons.

## Introduction

Within the social-cognitive approach (Bandura, 1986), social comparisons are related to a psychological mechanism that influences people’s judgment and behavior (Corcoran et al., 2011; Liu and Lan, 2016). In general, there are two types of social comparisons, upward and downward. Social comparison among peers can reflect individuals’ perceptions of others as “better or worse-off” than they are (Chua and Chang, 2016). When upward comparison (i.e., comparing oneself with those who are better) occurs in writing, individuals may want to improve themselves; this will lead to self-editing to compensate for their weaknesses. If downward comparison (i.e., comparing with those who are worse) occurs and individuals feel dissatisfied with others’ writing, they may think of other expressions to improve the writing, then edit their own manuscripts accordingly (Dijkstra et al., 2008; Calkin, 2018). A previous study indicated that, through comparison with peers, an epistemic belief is formed that can enhance one’s writing skills and abilities (Wright et al., 2019). However, most teachers adopt upward comparison in composition writing classes, with few using downward comparison to promote students’ writing skills. Accordingly, comparison of upward and downward social comparison (DSC) in Chinese composition writing (CCW) is the topic of interest of this study. In this study, students in the experimental group of upward social comparison (USC) learned from superior writing examples, while students in the experimental group of DSC learned by critiquing and correcting inferior writing examples. USC is described as making comparison with those who are better at something than we are, which may cause anxiety. On the other hand, DSC is related to making comparisons with those who are worse at something, and this can enhance our self-confidence (Saiphoo and Want, 2018). In Taiwan, CCW courses for junior high school students are required, in which the students need to complete 2 h of writing tasks each week. The effects of USC and DSC on these students’ CCW have not as yet been extensively studied, and so are the focus of this study.

Drawing on a social-cognitive perspective (Bandura, 2001), Dewaele and MacIntyre (2014) defined affect as the emotional interpretation of language learning experience. This interpretation can be further specified based on the achievement emotions theory (Pekrun, 2006); specifically, activating emotions such as hope, confidence and enjoyment are presumed to facilitate the use of flexible learning strategies, and can thus have a positive influence on learning performance. Conversely, deactivating emotions, such as anger, anxiety, and shame, imply detrimental effects on performance (Pekrun et al., 2011). That is, emotions are deeply implicated in the process of language learning by hampering or facilitating language development (MacIntyre and Vincze, 2017). However, many teachers teach writing based on example-based learning (Renkl, 2014; Lin et al., 2022), because example-based learning can facilitate students’ writing (Hoogerheide et al., 2019). Using examples for learning requires a few cognitive resources (Payne, 2012), such as time, mental effort, and conscious attention. These cognitive processes can activate or deactivate emotions by social comparison (Guastella and Dadds, 2008). Moreover, Lee and Evans (2019) found that the more USC that people engage in, the higher the levels of anxiety they deactivate, while Chatzisarantis et al. (2016) found that when students engage in DSC, they tend to persist with tasks and report activating levels of self-confidence after receipt of negative feedback from peers. However, in the discussion of activators, self-confidence enhancement, deactivators, and cognitive anxiety in social comparison, few studies have considered the effects on CCW; therefore, the purpose of this study was to explore how students’ cognitive anxiety and self-confidence enhancement interacted with their learning progress in CCW by adopting different social comparison approaches.

Integrating information technologies such as blogs can extend learning beyond the classroom, leading to an exchange of a variety of viewpoints that may affect the level of personal engagement with an issue (Levy et al., 2015; Marvell, 2018). Blogs can be used in an educational setting for a variety of purposes; for example, they can be used as a method for teaching written accuracy and personal reflection (Sackstein, 2015; Cumming et al., 2016). In line with this, the present study employed blogs for students to practice social comparison by making remarks about the superior or inferior examples of CCW posted by the teacher. Moreover, based on the achievement emotion theory, participants’ self-confidence enhancement and cognitive anxiety were examined in terms of six themes of writing practice. It is hoped that the results of this study can be used by CCW teachers to promote their students’ CCW performance.

## Literature review

### Cognitive anxiety in social comparison

Anxiety can affect cognitive performance through impairing attentional control (Eysenck et al., 2007). That is to say, cognitive anxiety is a transient condition characterized by tension and apprehension, coupled with activation of the autonomic nervous system (Spielberger, 1985). Cognitive anxiety may be associated with using maladaptive coping strategies, such as avoidance and withdrawal, in a stressful situation (Lysaker et al., 2005). If cognitive anxiety increases, this could be problematic, since cognitive function plays a major role in task performance (Lindgren et al., 2020). For example, in a test, increasing cognitive anxiety will decrease test performance (Thomas et al., 2017). Particularly, cognitive anxiety has been associated with lower verbal processing speed, but not with memory and planning functions or general neurocognitive performance that decrease writing performance (Stouten et al., 2017).

Cognitive anxiety consists of apprehension difficulties, concentration difficulties, lack of control over thoughts, and worry about the outcome (Fergus and Wheless, 2018). In social comparison, students may be concerned that their peers will outperform them, thus raising anxiety in their learning, but the results remain inconclusive (van Gennip et al., 2009). Individuals under age 18 tend to compare themselves with their friends (Callan et al., 2015); however, there is limited research related to cognitive anxiety in Chinese writing for junior high school students. Therefore, students’ cognitive anxiety related to their composition writing performance was explored in this study.

### Self-confidence enhancement in social comparison

The key aspect of confidence is that when an individual judges a cognitive task, it will provoke immediate reflection on his/her current performance. Within a learning task, confidence enhancement is positively related to cognitive and behavioral strategies (Marsh et al., 2012) and is directly relevant to task performance (Kleitman and Stankov, 2007). In using information technology, Yang (2009) stated that students who used blogs to learn found that their ability to communicate with peers helped them to critically reflect on the subject materials and enhanced their self-confidence. Moreover, students aged 15–17 have been described as being in a unique period of their life during which intensified social comparison related to their self-knowledge and self-evaluation affects their self-confidence (van der Aar et al., 2018). Thus, the extent to which social comparison could enhance junior high school students’ self-confidence after several rounds of composition writing was explored in this study.

## Research hypothesis

In the context of the social comparison theory (Festinger, 1954), researchers have extensively studied the relation between students’ achievement in the context of social comparison. Once established, USC enables learners to compare the level of a partner’s knowledge and to seek better learning approaches (Sangin et al., 2011). Moreover, Schwabe et al. (2019) claimed that providing opportunities for upward comparison will enhance students’ ability, whereas possibilities for downward comparison will decrease their ability. Previous research has pointed out that USC processes may explain an underlying mechanism of the negative effects of learning achievement (De Vries and Kuhne, 2015). That is, a negative relation between students’ DSCs and ability improvement does not only exist in specific domains, but also in different age groups (Marsh et al., 2014). However, the effects of different types of social comparison on the learning progress of CCW has not yet been studied. Thus, to understand how the writing progress can be improved via three types of social comparison was hypothesized as described below.

Neugebauer et al. (2016) argue that less knowledgeable learners who rely on social comparisons will be motivated to increase their engagement to learn knowledge from partners who are more knowledgeable. Moreover, when comparison options are restrained (i.e., only superior or only inferior examples are available to compare with), learners tend to seek comparisons that may effectively motivate them to achieve their learning goals (Ray et al., 2013). Conclusively, the review of others’ writing can help students to optimize their learning, and mirrors a form of learning progress (Wiliam, 2007; Clark, 2012). In this respect, writing progress is assumed to be a result of different types of social comparison. Therefore, hypothesis 1 was proposed as follows:

**H1:** Students’ writing performance will improve differently according to the three types of teaching methods.

Social comparison significantly influences self-evaluations and affective reactions (Zell and Strickhouser, 2020). Previous studies have suggested that the presence of confidence echoes the effects of an individual’s ability (e.g., Stankov et al., 2012a,b). Herron et al. (2019) used examples with video simulation to deepen students’ understanding of the learning content, and found that the use of examples as simulation-based learning can enhance students’ self-confidence. Moreover, the mindset of self-confidence has been found to affect language learning through social experience (Lou and Noels, 2020). For example, Theising et al. (2014) investigated how students’ self-confidence impacts their attitude toward peer feedback in a language class. In this regard, the question of whether or not the degree of self-confidence would vary as the practice times increased in the three approaches of writing composition was explored. Thus, hypothesis 2 was proposed as follows:

**H2:** Students’ self-confidence enhancement in Chinese composition writing (SCECCW) will improve differently according to the three types of teaching method.

The Anxiety and Uncertainty Management theory (AUM; Gudykunst, 2005) provides a framework to approach this question by describing the cognitive and affective challenges, including anxiety, that students are likely to experience during interactions with peers. Previous analyzes indicated that higher anxiety was related to USCs (Butzer and Kuiper, 2006). According to the social comparison theory, experiencing cognitive anxiety would inhibit students’ performance (Morony et al., 2013; Bum and Shin, 2015). As the students were taught with three different learning approaches to write compositions, their cognitive anxiety was expected to vary due to the different degrees of social comparison they engaged in. Therefore, hypothesis 3 was proposed as follows:

**H3:** Students’ cognitive anxiety in Chinese composition writing (CACCW) will improve differently according to the three types of teaching method.

Martens et al. (1990) supported the negative relationship between cognitive anxiety and self-confidence, and characterized self-confidence as the relative lack of cognitive anxiety. Moreover, Dickhäuser (2005) found that students’ self-concept related to anxiety was affected differently by external and internal comparison processes. Additionally, self-confidence is relevant to learning outcomes, and other key factors within the learning setting (Bum and Shin, 2015). As the primary purpose was to examine the effects of social comparison on self-confidence and anxiety (Seaton et al., 2010), different types of social comparison were combined to explore the benefits to the CCW learning outcomes. Taken together, how different types of reflection would interact with cognitive anxiety and self-confidence enhancement in students’ writing progress was hypothesized as H4:

**H4:** The difference in the Chinese writing progress of students learning with the three types of teaching method is impacted by their SCECCW and CACCW.

## Research design

### Participants and procedure

In this study, we adopted purposive sampling to select students from one urban junior high school located in Taipei city (note that compulsory education is up to ninth grade, year 3 of junior high school in Taiwan). Six ninth-grade classes consisted of 181 students in the target school. Because ability grouping is prohibited in the junior high school system in Taiwan, there was no difference in the writing ability of the students in each class, because they were normally distributed as they entered the school according to school policy. After asking the Chinese language teachers to assist with this research, one teacher was willing to participate; she taught three classes with a total of 90 students (*M* = 14.47 years old) who were selected for this study. These 90 students were randomly divided into three groups: 31 were placed in the NCG, 32 in the UCG, and 27 in the DCG. All three groups had composition lessons twice a week (45 min per lesson). The students all had previous experience of using blogs. Before the beginning of the research, the teacher taught the UCG and DCG students how to use the blog for reading, and how to give responses or comments using a mobile device.

Totally six lessons were taught with a different topic chosen for each. The composition topics included “Presents,” “A song I like,” “At the swimming pool,” “The person I want to be,” “This time, I will be different,” and “Those whom I treat as friends.” All the topics were written as narrative prose.

The CACCW questionnaire was given to the students before each trial, a total of five times. The SCECCW questionnaire was given to the students after each trial, that is, also five times in total. In the preface of the questionnaire, the participants were notified that they were participating in an evaluation study, that the data they provided was anonymous and that the study might be published. Additionally, we also collected and recorded information on the students’ writing performance for the five different compositions.

### Experiment setting

Blogs have been recognized as a valuable, flexible and easy-to-use web 2.0 technology tool for teaching and learning (Halic et al., 2010; Ifinedo, 2017). According to Yang (2009), the study found that students’ communication ability and reflection of learning has been enhanced by using blogs for learning. Researchers found that students recognize that blogs are a useful tool for their learning (Halic et al., 2010; Chen et al., 2015; Ifinedo and Usoro, 2016). In light of this, the present study focused on implementing social comparison, and used blogs as a writing and interactive tool for students to refer to compositions, and post comments on the writings. Three intact classes of students were designated as the three groups: the first class was the no comparison group (NCG), the second class was the upward comparison group (UCG), and the third class was the downward comparison group (DCG). These three classes were taught Chinese by one teacher. Particularly, the UCG and DCG students had to use the blogs to read some anonymous writings and to give their responses to or comments on the provided samples which had been evaluated by teachers as best or worst writing examples. Briefly, the features of the three groups are elaborated as follows.

The NCG: This was the control group. In this group, the teacher announced the writing topics and explained the meaning to the students. The NCG Students had to make a plan of their writing structure before they started to write.

The UCG: This was one of the two experimental groups. In this group, students first received five superior writing samples which were related to the same writing prompt. The UCG students had to give their feedback on the samples, including the reasons why they were considered superior. They received no input from the teacher at this stage. They had to mark what parts of the writing they preferred, and they had to post their comments and take on the Blog that the teacher regulated. The teacher then responded with additional remarks to point out which of the student’s comments were correct, useful or appropriate.

The DCG: This is the other experimental group which was taught to reflect on five samples of inferior compositions which the teacher posted on the blog. The DCG students also received a message telling them which post they had to read and give their responses to or comments on. The DCG students marked those parts they thought might be incorrect as well as mistakes they found in the examples. In other words, the students conducted peer assessment based on the criteria which had been taught by the teacher beforehand, and highlighted the problematic sentences in each sample. They were asked to revise as many as possible in each sample without any feedback from the teacher, but they had to post their comments to the blog for the teacher to understand how much effort they had put into the task.

**FIGURE 1:**
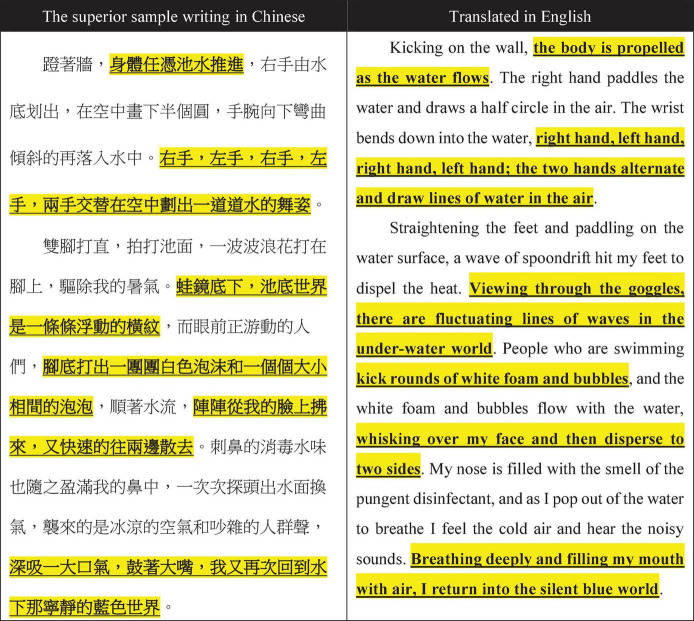
Example of a student’s critique in the upward comparison group.

The topic of the sample writing was the same for the UCG and DCG: describing people swimming in a pool. These sample writings were written by students in the same grade but who did not take part in this research. After grading by the teachers, the writings were divided into groups and the superior and inferior samples were posted separately on the blog. The superior sample shown in Figure 1 features the use of some exquisite, ornamental language and some rhetorical skills that the students could learn from. A student in the UCG highlighted (in yellow) what he/she considered to be good sentences in the sample writing that he/she could not think of him/herself. The students could apply such words or sentences in their own writing on the same type of topic.

**FIGURE 2:**
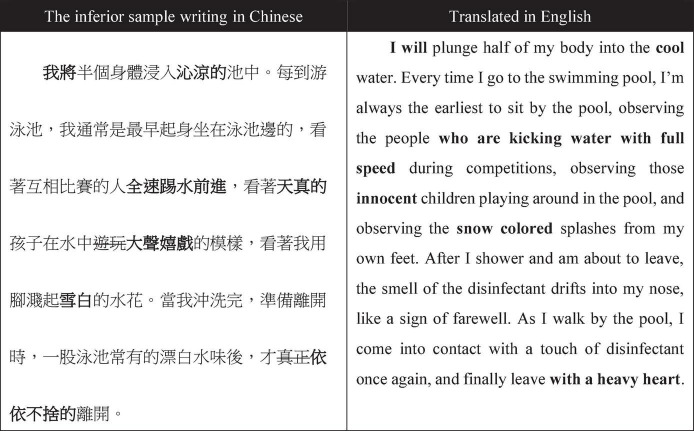
Example of a student’s critique in the downward comparison group.

On the other hand, students in the DCG were given an inferior writing sample which also described people swimming in the pool. The DCG students were asked to improve the sample writing using some rhetorical skills such as similes, metaphors, and metonymy. Figure 2 shows an example of a DCG student’s revision of an inferior composition (the revisions are marked in bold in the original Chinese text and in the English translation). From the revision of Figure 2, the present study can see that the student was trying to add some adjectives to the original sentences.

### Teachers’ assessment of students’ performance

The present study used a writing performance assessment rubric which referred to the Competence Indicators of Mandarin Writing (Ministry of Education, 2010) to ensure the effect of the writing performance assessments. It also focused on some parts of language learning such as grammar, writing style, vocabulary usage, language accuracy, coherence, and fluency as previously mentioned.

#### The assessment rubric

The CCW assessment rubrics are divided into six levels. To gain 6 points, the following criteria should be met (Chang and Sung, 2019): (1) Content: Appropriate information is included with reference to the topic and theme, and is elaborated with details to meet the objective; (2) Organization: The composition is complete, well-structured, and coherent; (3) Vocabulary and Sentence Structure: The use of vocabulary and phrases is precise and accurate with an effective application of a wide range of sentence structures for fluent expression; (4) Chinese characters, Format, and Punctuation: Spelling, format or punctuation is almost flawless. The superior examples of CCW posted by the teacher should have scored 6 points, while the inferior examples only scored 1 or 2 points.

#### Credibility of assessment

For triangulation purposes, three Chinese language teachers who clearly understood the six composition grading criteria, which were the same as the composition grading criteria of the Comprehensive Assessment Program for Junior High School Students in Taiwan, were invited to grade the writing performance of the study participants. All the students’ compositions were rated by the three raters, and the rating results were compared and rationalized until they agreed on a score to ensure the consistency of the rating. The three raters repeated the above process until they achieved a consistent result above 95% based on Kendall’s coefficient of agreement analysis (Tong et al., 2020). If the coefficient was above.9, it shows that the appraisers were applying essentially the same standard when assessing the samples, which is considered as excellent.

## Measuring instruments

### Questionnaire item development

Self-confidence enhancement: Adapted from Grundy (1993), confidence level is related to the performance of a specific skill; moreover, it refers to a subjective cognitive experience which results in judgments of certainty relevant to one’s performance (Jackson and Kleitman, 2014). Cognitive anxiety measurement: Adapted from Viney and Westbrook’s (1976) Cognitive Anxiety Scale (CAS) is a means of measuring cognitive anxiety. Adapting from above researchers, this study designed the questionnaire by professionally translating the original items into Chinese, then using the forward-backward method to verify its face validity.

In this study, a 7-point Likert scale was used for the measurement, with 1 representing strongly disagree and 7 representing strongly agree. The suitability of the questionnaire items in each construct was tested by first-order confirmatory factor analysis (CFA) after the first trial. In the original questionnaire, there were 12 items in the CACCW construct, and 10 in the SCECCW construct.

According to first-order CFA, the χ^2^/*df* values of each construct were all less than the threshold value of 3. CACCW is 2.71 and SCECCW is 2.19, both of them are less than 3. In addition, the threshold value of the goodness of fit index (GFI) is 0.9 and 0.92 for CACCW and 0.94 for SCECCW are both above 0.9 and the threshold of the adjusted goodness of fit index (AGFI) is 0.9, and CACCW is 0.95 and SCECCW is 0.91, both of them were above the cutoff value of 0.90. The threshold value of the root mean square error of approximation (RMSEA) is 0.08 and CACCW is 0.76 and SCECCW is 0.42, and both of them were less than 0.08. The threshold of standardized root mean square residual (SRMR) is 0.05 and 0.05 for CACCW and 0.32 for SCECCW were less than 0.05. Above indicating that there was a good fit for each construct (Hair et al., 2009). According to the first-order CFA, seven items for the SCECCW and nine for the CACCW remained.

Moreover, the present study tested if the reliability of the two constructs was acceptable in terms of their Cronbach’s α value by using the software, SPSS 22. Subsequently, the questionnaire was applied to another four trials after the participants finished each composition. Self-confidence enhancement: How confident the participants felt compared to the first time they completed a writing task was measured after each writing (e.g., “Compare how confident you feel now to how you felt the last time you completed a writing task in terms of: how confident you are in using a variety of vocabulary for better writing; how confident you are in using language precisely in writing”). The Cronbach’s α = 0.87 and CR = 0.90, indicating that the internal consistency and composite reliability of this construct were acceptable. Cognitive anxiety measurement: students’ CACCW was measured after each writing, including, “During this writing task, I was anxious because I didn’t know how to get started.” The Cronbach’s α = 0.88 and CR = 0.91, indicating that the internal consistency and composite reliability of this construct were acceptable.

In addition, this study aimed to analyze the power of the factors influencing students’ composition writing performance progress. For the considered factors and interaction, the estimation of effects was carried out by ANOVA, analysis of variance, to investigate the significant differences among the three groups, and *post hoc* analysis was also performed to compare the difference among three groups.

## Results

The collected data were analyzed using SPSS 22 to test the significance of the four hypotheses.

### Serial analysis of writing performance progress, self-confidence enhancement in Chinese composition writing, and cognitive anxiety in Chinese composition writing

**FIGURE 3:**
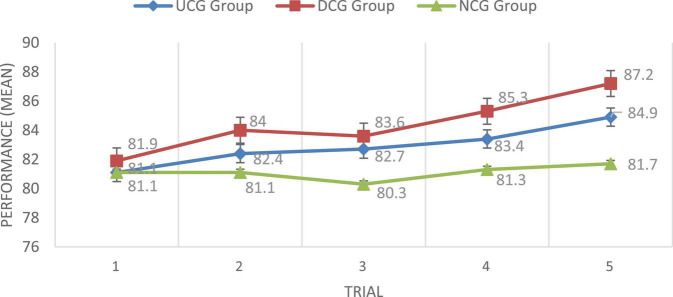
Writing performance progress for the three groups.

To assess the ability to learn from distributional information alone, a serial reaction time task can be categorized into a sequence of input stimuli (Hunt and Aslin, 2010). Serial analysis was adopted in this study to examine the composition writing performance progress through the SCECCW and CACCW of the three groups, NCG, UCG, and DCG, during the 5 weeks’ experiment. Regarding the performance in composition writing, Figure 3 revealed a series chart of the three groups’ composition writing performance. Compared to the UCG and NCG, the DCG had higher writing performance, while the NCG had the lowest performance among the three groups. Furthermore, the UCG and DCG students’ composition writing performance generally improved across the five trials (with the exception of the DCG from trial 2 to trial 3).

**FIGURE 4:**
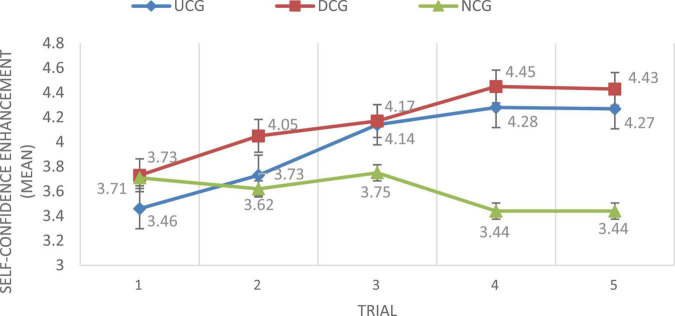
SCECCW for the three groups.

Figure 4 reveals the change over the five trials of the three groups’ participants’ self-confidence enhancement in composition writing. Compared to the UCG and NCG, the DCG had greater SCECCW, and the NCG had the lowest self-confidence enhancement among the three groups. In addition, the self-confidence of the participants in the UCG and DCG increased across the five trials, while the self-confidence of the NCG students decreased. Figure 4 shows that the SCECCW of the NCG decreased over the five trials, while the SCECCW of the UCG and DCG generally increased. The self-confidence level of participating students with upwards and downwards treatments all showed improvement over time.

**FIGURE 5:**
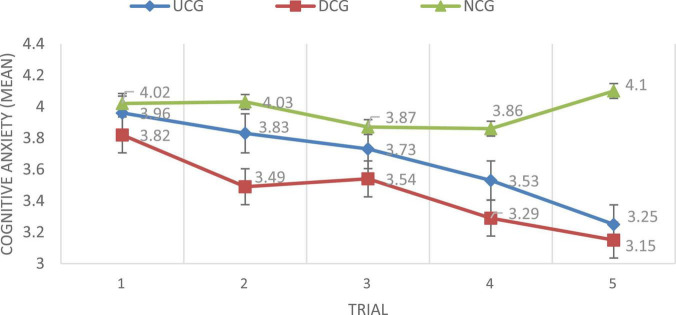
CACCW for the three groups.

Figure 5 reveals the change over the five trials of the three groups of participants’ cognitive anxiety in composition writing. Compared to the UCG and DCG, the NCG had higher cognitive anxiety, whereas the DCG had the lowest cognitive anxiety among the three groups. Moreover, the self-confidence of the participants in the UCG decreased across the five trials, while the self-confidence of the DCG students decreased along with the number of practice times (with the exception of trial 2 to trial 3). For the NCG, participants’ cognitive anxiety decreased from trial 1 to trial 3, and increased from trial 3 to trial 5 to be higher than it was in trial 1.

### The difference between groups in relation to writing progress, self-confidence enhancement and cognitive anxiety

The present study investigated the differences among the three groups in relation to writing performance progress, SCECCW, and CACCW in this study. Table 1 shows the results of the repeated measures ANOVA and *post hoc* comparison. Means of writing performance progress, SCECCW, and CACCW are significantly different in the three groups. For the writing performance progress, *F* (_2,87_) = 3.36 (*p* < 0.05) which indicates that there is a significant difference in the three groups, and the means of the UCG (*M* = 3.85) and the DCG (*M* = 5.38) are higher than the means of the NCG (*M* = 0.80). For SCECCW, *F* (_2,87_) = 6.04 (*p* < 0.01) which indicates that there is a significant difference in the three groups, and the means of the UCG (*M* = 3.98) and DCG (*M* = 4.17) are higher than that of the NCG (*M* = 3.59). For CACCW, *F* (_2,87_) = 4.62 (*p* < 0.01) which indicates that there is a significant difference among the three groups, and the mean of the NCG (*M* = 3.98) is higher than both the UCG (*M* = 3.66) and DCG (*M* = 3.45). Regarding the degree of writing performance progress, participants in the UCG and DCG showed greater improvement than those in the NCG. In a similar way, participants in the UCG and DCG also exhibited a higher level of SCECCW than those in the NCG. In addition, the present study found that the students in the UCG and DCG had a significant decrease in their CACCW levels than those in the NCG.

**TABLE 1:** Summary of writing progress, self-confidence enhancement and cognitive anxiety.

Variable	Group	N	Mean	*SD*	*F*	Post hoc comparison
Writing progress	NCG	31	2.80	1.31	3.36*	DCG, UCG > NCG
	UCG	32	3.85	1.53		
	DCG	27	5.38	1.31		
SCECCW	NCG	31	3.59	0.59	6.04**	UCG, DCG > NCG
	UCG	32	3.98	1.46		
	DCG	27	4.17	1.23		
CACCW	NCG	31	3.98	0.86	4.62**	NCG > UCG, DCG
	UCG	32	3.66	1.24		
	DCG	27	3.45	0.93		

**p < 0.01, *p < 0.05.

## Discussion

Based on the achievement emotion theory, this study investigated participants’ self-confidence and cognitive anxiety change along with the upward and DSCs. The results of this study indicate the effects of three different forms of social comparison. Firstly, the students in the UCG and DCG showed greater improvement in CCW than those in the NCG. Secondly, the students in the UCG and DCG showed greater improvement in SCECCW than those in the NCG. Thirdly, the students in the UCG and DCG experienced a greater reduction in cognitive anxiety about CCW than those in the NCG. If we only compare between the UCG and DCG, the analysis result shows that there has no significance between the two groups. The verification of the research hypotheses is presented as follows.

**H1:** Students’ writing performance will be improved differently according to the three types of teaching methods.

When comparison options are restricted (i.e., only superior or only inferior examples are available for comparison), learners tend to seek comparisons that may effectively enhance themselves to achieve their learning goals (Ray et al., 2013). However, these studies did not examine writing improvement in relation to the effect of number of practice times in using Blogs. In addition, previous research has found that writing performance will be significantly improved with more practice (Oppenheimer et al., 2017). Supporting this point of view, the current study revealed that when the students examined other works, they could make further revisions of their text development, writing organization, and writing style. The results of the present study indicate that no matter which group the participants were allocated to, their writing performance improved, and furthermore, participants in both the UCG and DCG may have had a better cognitive process when they had more composition writing practice. This result is partially consistent with some previous studies which indicated that USCs can trigger people and improve performance through increased practice (Yang, 2010).

**H2:** Students’ SCECCW will be improved differently according to the three types of teaching method.

According to the social comparison theory, students’ anxiety level would be promoted or inhibited as a result of upward comparisons (Johnson and Stapel, 2007). When less knowledgeable learners practice upward comparison, it can increase their anxiety. Besides, seeing one’s own potential in the superiority of others may turn into positive affect about oneself (Neugebauer et al., 2016). Moreover, Takahashi et al. (2009) and Swencionis and Fiske (2014) proposed that downward comparisons can produce many kinds of emotional responses and show different activations in the state anxiety circuitry. Findings of this study showed that the NCG participants experienced a higher degree of CACCW as the number of practice times increased. Regarding cognitive anxiety as the deactivated factor of achievement emotion theory, the results also showed that given platitudinous practice time, with social desirability (Gruda and Hasan, 2019), participants’ cognitive anxiety as state-anxiety deactivated at a much more substantial rate in both the UCG and DCG than it did for the students in the NCG.

**H3:** Students’ CACCW will improve differently according to the three types of teaching method.

Some people would like to show their superiority to others in downward comparisons (Wills, 1981) to make themselves feel prominent and outstanding (Larrick et al., 2007), and also to enhance their self-confidence (Brown et al., 2007). A previous study used examples with video simulation to deepen students’ understanding of the learning content, and found that it can enhance students’ self-confidence (Herron et al., 2019). Moreover, Theising et al. (2014) assessed how students’ self-confidence influences their attitude toward their reception of peer feedback throughout the experimental language curriculum. The results of this study in support of this point of view is that the DCG participants enhanced their self-confidence more significantly than the NCG participants by comparing their last practice to their first practice of CCW. Regarding self-confidence as the activating factor of achievement emotion, the results of the present study verified that the UCG participants demonstrated a greater activation of SCECCW than the NCG participants as a result of the increased number of practice times.

**H4:** The difference in the Chinese writing progress of students learning with the three types of teaching method is impacted by their SCECCW and CACCW.

Within educational settings, lower confidence levels have been positively associated with higher levels of English and Mathematics anxiety (Stankov et al., 2012a; Morony et al., 2013). However, the anxiety-related experience might put one’s own achievements in doubt (Want and Kleitman, 2006). Additionally, self-confidence is relevant to learning outcomes, and other key factors within the learning settings (Bum and Shin, 2015). Dickhäuser (2005) found that students’ self-concept related to anxiety was affected differently by comparison processes. Students who used blogs for learning subjects by responding to or receiving comments have been found to be able to enhance their self-confidence (Yang, 2009; Bowman and Akcaoglu, 2014). The results of this study indicated that the UCG and DCG outperformed the NCG. The UCG and DCG students achieved better learning outcomes than the students in the NCG. Supporting the above study and responding to the question about whether there is a difference in the learning progress of the three groups, the present study revealed that students using the two types of comparison performed better in terms of their writing progress with less cognitive anxiety, and experienced better self-confidence enhancement than the students in the NCG. Briefly, the results of this study are based on series analysis, which is different from previous studies that focused on one-shot data collection and analysis, for example, Zhang et al.’s (2020) study which testified the motivation and demotivation with anxiety in one experiment. With time series data analysis, the activator of self-confidence will increase, but the deactivator of cognitive anxiety will increase as learning trials increases.

## Conclusion

In order to improve their writing performance, there is a need for students to adopt example-based learning to reflect on and enhance their writing abilities (Cheng and Chan, 2019). In this study, we used the social comparison theory to provide insights into these complex findings by examining the self-confidence, cognitive anxiety, and behavioral aspects related to CCW. The main goal of this adaptive educational approach was to create a social comparison environment by using blogs to support peer learning that can evoke learners’ dynamic cognitive and affective states. The results of this study indicated that providing superior or inferior examples to learners can foster their understanding of the principles and concepts, generate confidence, and eliminate anxiety in CCW; their learning of the CCW principles and concepts generate confidence, and reduce anxiety in CCW.

### Implications

People have a propensity to follow the norm of “seeing our own strengths and seeing others’ shortcomings” in peer assessment (van Boven et al., 2003). The results of this study support the premise that the writing performance of students can differ based on the characteristics of superior or inferior samples. The critical role of upward or downward comparison as an essential factor affecting students’ cognitive and affective perceptions has been highlighted in this study. Therefore, it is specifically recommended that teachers employ the upward and downward comparison instructional approaches of teaching composition writing.

It is useful for teachers to consider how individual students interact with various aspects of their social context in relation to their cognitive anxiety to apply different teaching approaches in writing compositions. Comparison of their own writing to superior or inferior writing samples may cause some students to feel more or less cognitive anxiety, which may affect their willingness to engage in writing. However, the results of this study indicated that the more practice they had, the less cognitive anxiety the participants experienced, except for those students in the NCG. Thus, we suggest identifying individual students’ inclination for social comparison, and applying downward comparison for more effective learning in composition writing.

### Limitations and future study

There are two limitations to this study: the sampling and the tool. First, we selected ninth graders in Taiwan as our sample in this study. According to Stankov et al. (2012b), an individual’s cognitive processes and affective states will change with age. We therefore suggest that future studies can expand the sample size across students of different grades and also from different schools to compare the differences in their social comparison across ages. Second, in this study, we used a blog as the online forum tool to support learning. Therefore, the influences of contemporary tools that students use for communication in efficient and popular ways (e.g., Facebook, LINE, Skype) should be studied further (Yen et al., 2015). Moreover, the difference between blogging and traditional paper-and-pencil delivery of the writing can also be further studied to compare the different effects of the two moderating approaches.

## Data availability statement

The raw data supporting the conclusions of this article will be made available by the authors, without undue reservation.

## Ethics statement

Ethical review and approval was not required for the study on human participants in accordance with the local legislation and institutional requirements. Written informed consent to participate in this study was provided by the participants’ legal guardian/next of kin.

## Author contributions

J-CH: original draft. K-HT: data analysis and review and editing. M-YH: review and editing. C-YL: data collection. All authors contributed to the article and approved the submitted version.
